# The Queen's Jubilee Hospital

**Published:** 1905-12-02

**Authors:** 


### THE QUEEN'S JUBILEE HOSPITAL.

### MANY MORE RESIGNATIONS.

We are informed that three members of the visiting staff of the Queen's Jubilee Hospital have resigned, as well as the matron, sister, and staff-nurse. The wholesale resignations of the staff, which occurred at the same hospital early in this present year will be remembered. In all the circumstances every disinterested person must feel that the present administration of the affairs of this Institution cannot much longer endure if it is to continue to exist as a Metropolitan charity.

